# Hypometabolism of the left middle/medial frontal lobe on FDG‐PET in anti–NMDA receptor encephalitis: Comparison with MRI and EEG findings

**DOI:** 10.1111/cns.14125

**Published:** 2023-02-23

**Authors:** Chenpeng Zhang, Yong Hao, Gan Huang, Mei Xin, Shuwei Bai, Yangtai Guan, Jianjun Liu

**Affiliations:** ^1^ Department of Nuclear Medicine, Ren Ji Hospital Shanghai Jiao Tong University School of Medicine Shanghai China; ^2^ Department of Neurology, Ren Ji Hospital Shanghai Jiao Tong University School of Medicine Shanghai China

**Keywords:** 18F fluorodeoxyglucose, anti‐N‐methyl‐D‐aspartate receptor encephalitis, magnetic resonance imaging, positron emission tomography

## Abstract

**Objectives:**

To investigate changes in brain‐glucose metabolism in anti‐N‐methyl‐D‐aspartate receptor (NMDAR) encephalitis, and compare results with MRI and electroencephalography (EEG) findings at different disease stages.

**Methods:**

The clinical data of 18 patients (median age, 35 years; 11 men) were retrospectively collected. Patients were divided into groups based on the time of symptom onset to examination, (≤1 month, >1 but ≤3 months, >3 months). Two‐sample *t*‐test results were compared with age and sex‐paired healthy controls using statistical parametric mapping and verified using a NeuroQ software normal database with a discriminating z‐score of 2.

**Results:**

Abnormal patterns on FDG‐PET differed over time (*T* = 3.21–8.74, *Z* = 2.68–4.23, *p* < 0.005). Regional analysis showed hypometabolic left middle or medial frontal cortex in 4/5, 5/7, and 5/6 patients, respectively. Time‐subgroup analysis revealed hypermetabolic supertemporal cortex in 4/5, 5/7, and 2/6, patients, respectively. MRI and EEG abnormalities in any region and stage occurred in 10/18 and 10/16 patients, respectively. MRI and EEG time‐subgroup analysis showed abnormalities in 5/9, 4/5, and 1/4, and 1/3, 6/7, and 3/6 patients, respectively. Abnormal temporal lobes were detected most frequently in MRI analyses and occurred in 3/10 patients.

**Conclusions:**

Decreased left middle/medial frontal metabolism could be common to all stages. Metabolism in other regions, MRI, and EEG results were associated with the progression of anti‐NMDAR encephalitis. The sensitivity rate of FDG‐PET was superior to that of MRI and EEG.

## Graphical abstract

Hypometabolic left middle/medial frontal cortex are possible FDG‐PET metabolic patterns in more than half of patients with all stages of anti‐NMDAR encephalitis. Patient MRI and EEG examinations were optimal at 2–3 months of symptom onset. Evaluation with FDG‐PET could be superior to MRI and EEG in patients with anti‐NMDAR encephalitis.

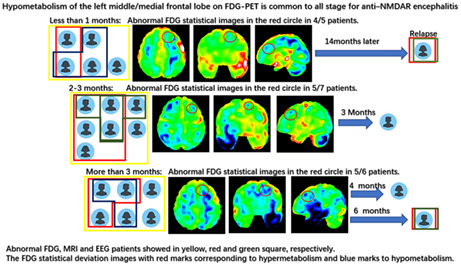

## 1 INTRODUCTION

Anti‐N‐methyl‐D‐aspartate receptor‐autoimmune encephalitis (NMDAR‐AE) is the most common type of AE. Fortunately, NDMAR‐AE can be treated with immune‐related therapies that significantly improve prognosis.^1^ This disease presents various complex neural and mental symptoms that often lead to a delay in diagnosis.^1^, ^2^ The diagnostic criteria mainly depend on detecting antibodies in serum and cerebrospinal fluid (CSF), and monitoring immune response to treatment.^1^, ^3^ However, these methods are invasive and are often not applicable during early evaluation and management.

FDG‐PET and MRI examinations are important and recommended for auxiliary diagnosis of NMDAR‐AE.^1^, ^3^, ^4^, ^5^, ^6^, ^7^, ^8^, ^9^, ^10^, ^11^, ^12^ Previous studies showed that FDG‐PET provided earlier detection and had a higher diagnostic sensitivity rate than MRI.^4^, ^5^, ^6^, ^13^, ^14^, ^15^, ^16^, ^17^ However, there is no consensus on the abnormal patterns of FDG‐PET, including hypo or hypermetabolism of the frontal, temporal, and occipital lobes, and the basal ganglia and cerebellum. To the best of our knowledge, most studies have been case reports or included fewer than five patients per group with subgroup analysis,^4^, ^5^, ^6^, ^7^, ^8^, ^9^, ^12^, ^13^, ^14^, ^15^, ^16^, ^17^, ^18^, ^19^, ^20^ causing confusion regarding the accurate diagnosis of the disease. Electroencephalography (EEG) is useful for auxiliary diagnosis and evaluation of prognosis.^21^, ^22^, ^23^, ^24^

This study focused on the changes in FDG‐PET results with disease progression in anti‐NMDAR encephalitis and compared the clinical auxiliary diagnosis with MRI and EEG results at different stages of the disease.

## 2 MATERIALS AND METHODS

### 2.1 Clinical data

Patients who had undergone hospitalization for suspected encephalitis and ^18^F‐FDG‐PET/CT at our hospital were retrospectively selected. The exclusion criteria were as follows: (1) patients with other types of encephalitis, such as viral encephalitis, herpes simplex viral encephalitis, Hashimoto's disease, and mental illness and (2) AE according to consensus clinical diagnostic criteria^3^ but not of the NMDAR type.

This retrospective cohort study was approved by our institutional review board, and the requirement for obtaining informed consent was waived.

Clinical data considered potentially relevant to AE were collected from patient medical records. Data included age at FDG‐PET/CT imaging, sex, clinical history, CSF and serum antibodies, brain MRI scans, EEG, FDG‐PET/CT treatment, and tumor histology within at least the previous 5 years from disease onset. The subgroup analysis was based on the timing of PET scans after disease onset three groups: ≤1 month, >1 but ≤3 months, >3 months, which represent acute, subacute, and chronic phase. All patients were stable at the time of imaging without the use of sedatives or tranquilizers.

### 2.2 Neuronal antibody measurement

All patients with NMDAR‐AE underwent serum and CSF antibody tests. Serum and CSF immunoglobulin titers were measured using cell‐based and dot immunobinding assays.

### 2.3 Acquisition of ^18^F‐FDG‐PET and MRI data

Whole‐body PET acquisition began approximately 45 ± 10 min after intravenous administration of approximately 37 MBq/kg of FDG, and brain imaging was performed for 10 min. PET/CT scans were performed in 15 patients using a Siemens mCT scanner (Siemens Healthcare) equipped with lutetium oxyorthosilicate (LSO) detectors and a 64‐slice CT. These PET images were reconstructed using TrueX (an ordered subset expectation maximization [OSEM] iterative reconstruction). Alternatively, PET studies were performed on three patients using a uMI®780 scanner (United Imaging) with high‐resolution digital PET and 160‐Slice ultra‐fast CT. These PET images were reconstructed using time‐of‐flight (TOF) OSEM iterative reconstruction. All patients in our center fasted for at least 6 h before the FDG injection. Patients waited in a relatively quiet and bright environment and did not wear an eye mask while undergoing PET/CT.

MRI sequences were acquired at our hospital and included T1‐weighted, T2‐weighted, T2‐weighted fluid‐attenuated inversion recovery (FLAIR), DWI sequences, and contrast‐enhanced MRI in sagittal, coronal, and transverse views.

The reported routine‐awakening EEG data were identified as normal or abnormal based on patient medical records.

### 2.4 Visual assessment of FDG‐PET/CT and MRI

Visual assessment of PET images was performed separately by two experienced nuclear‐medicine physicians who were aware that the patients had suspected encephalitis, but did not have any information from other examinations such as MRI and EEG in advance. Regions with metabolic changes compared to the contralateral were visually identified as abnormal, and bilateral lobe metabolism changes were compared to the cerebral cortex using maximum intensity projection. The cerebellar metabolism is slightly lower than that of the cerebral cortex, and abnormal hypermetabolism occurs when the cerebellar metabolism is equal to or exceeds that of the cerebral cortex.

Two well‐trained radiologists who were blinded to the PET information were involved, and identified hyperintense lesions on T2, FLAIR, or DWI images, with the exclusion of other nonspecific causes such as age‐related white‐matter hyperintensities, lacunar infarction, and brain atrophy.

Electroencephalography data were retrieved from the medical records.

### 2.5 Software analysis of FDG‐PET/CT images

Considering age and sex differences in brain FDG metabolism, we initially used a fully‐paired control to create a healthy normal database that determined possibly‐related brain regions by statistical parametric mapping (SPM) analysis to make a voxel‐wise comparison of anisotropy in the whole brain. The voxel‐wise analysis for glucose metabolism was analyzed by counting the (18)F‐FDG activity of each hemisphere on the normalized and spatially smoothed PET images, and the number of voxels with significant hypermetabolism and hypometabolism. To expand the scope of the brain region adaptations, we validated with a NeuroQ software open database and patient analysis to calculate the sensitivity rate.

Before statistical analysis, all images were spatially normalized into a standard brain space by global grand‐mean scaling and smoothed using SPM‐8 in a MATLAB R2018b environment (MathWorks, Inc.). Following validation, FDG‐PET processing was performed based on an optimized semiquantitative procedure (details described above).^25^, ^26^ Next, images were tested for relative whole‐brain hypo and hypermetabolism using a two‐sample t‐test implemented in SPM that compared the AE group with a group of age and sex machine‐paired healthy individuals without any neurologic or psychiatric diseases. Because only three patients were scanned with a different apparatus, we used machine‐matched healthy individuals as controls, and the different PET scans were not entered as a nuisance covariate. The resulting SPM‐t maps, with a threshold at *p* < 0.005 ( *k* = 50), represented the basis for the assessment of regional hypo and hypermetabolism.

NeuroQ implanted in Philips IntelliSpace Portal software v7.0 (Philips Healthcare) was used to process ^18^F‐FDG‐PET/CT brain images. After automatic rigid deformation, the entire brain was divided into 47 regions. The metabolism of each region was calculated by comparison with the default software database. Areas of hypo or hypermetabolism in the brain differing from the mean were recorded as abnormal using a cutoff z‐score value of 2.0.

## 3 RESULTS

### 3.1 Patient characteristics

A total of 53 patients with suspected encephalitis who underwent PET‐CT at our hospital between January 2015 and March 2021 were retrospectively identified. Eighteen patients were included in the analysis (Figure S1 shows the flow diagram). Patient characteristics (median age, 35 years; range, 14–67 years) and other clinical information are summarized in Table 1. The Data of patients' age exhibited a normal distribution (*p* = 0.73). The results of healthy controls were compared with age and sex‐paired patients.

**TABLE 1:** The individual characteristics and clinical data of patients with NMDAR AE.

Pat.	Gender	Age (years)	Symptoms at hospitalization	Therapy	PET therapy duration^a^	Follow‐up duration^b^	Relapse
1	M	35	Cognitive impairment, tremor in both upper limbs, gatism, weakness of both lower limbs for 1 month	IVMP	42	133	‐
2	M	63	Dizziness, insomnia, limb paresthesia for 1 year,	IVIg	−3	266	‐
3	M	58	Fever with nausea, dizziness, headache for 50 days, somnolence, cognitive impairment	IVMP, PLEX	2	956	‐
4	F	14	Cognitive impairment and paroxysmal whole body shaking for 3 month, depressed, epilepsy	IVMP, PLEX	3	666	‐
5	M	56	Unconsciousness epilepsy third for 1‐month, cognitive impairment for half a month	IVMP, IVIg, PLEX,	9	295	‐
6	F	24	Speech disorders accompanied by abnormal behavior for half a month	IVMP, IVIg,	2	101	‐
7	M	34	Headache and fever for more than a week, seizure once	IVMP	−2	255	‐
8	F	35	Blurred vision for 3 months, weakness in the right limb for 2 months, and aggravation with slurred speech for 1 month; hallucinations, impairment of orientation, and decreased levels of consciousness recently	IVMP, IVIg,	9	139	‐
9	M	39	Headache for more than 1 month accompanied with raving for 5 days	IVMP, IVIg,	34	461	‐
10	M	27	Numbness in the right side of the body, fatigue for more than 2 months, slurred speech, drooling, hearing loss, dyspepsia, walking instability, mood disorders, memory loss	IVMP, IVIg,	17	173	‐
11	M	21	Headache more than 10 days, seizure once 2 days ago	IVMP	4	202	‐
12	M	67	Speech disorders accompanied by abnormal behavior, gatism, cognitive impairment for 50 days	IVMP, IVIg	4	97	‐
13	F	17	Unsteady walking with bilateral temporal visual field defect for more than 5 months	IVMP, IVIg,	3	226	4 months later
14	F	20	Paroxysmal loss of consciousness after high fever, visual and auditory hallucinations, seizure	IVMP	15	265	14 months later
15	F	35	Pain in the posterior neck and posterior brain area, slight dizziness, accompanied by nausea for 5 days	IVMP	4	137	
16	M	23	Headache and fever for 2 weeks with psychiatric symptoms, visual and auditory hallucinations for 6 days	IVMP, IVIg, PLEX	30	325	3 months later
17	F	48	Cognitive impairment, Speech disorders accompanied by abnormal behavior (e.g., 10 days of incomprehensible talk)	IVMP, PLEX	13	160	
18	M	53	Legs twitch for 1 year, dizziness with diplopia for half a year	IVMP, IVIg, PLEX	0	325	6 months later

*Note*: No. 1 had a history of viral encephalitis of 5 years. No.3, No.8, and No.12 had a history of viral encephalitis of 3 months. No.6 diagnosed as ovarian teratoma. No.12 History of surgery for liver cancer, no sign of recurrence. No. 13 had ovarian endometriosis diagnosed by pelvic MRI+. IVMP, intravenous methylprednisolone. IVIg, intravenous gamma globulin. PLEX, plasma exchange.

^a^ Days: The time from the onset of therapy to PET scan.

^b^ Days: The time from the onset of symptoms to the last contact.

Four patients relapsed during follow‐up in this study, three of which were performed the PET after more than 2 months (178, 69, and 162 days) and treated after 3 months (181, 99, and 162 days) from symptom onset, and relapsed 3–6 months later (pat.13, pat.16, and pat.18). Another patient performed the PET at 30 days, treated at 45 days and relapsed at 14 months (pat.14). The earlier the PET examination, the later the recurrence.

A review of medical history revealed that in total, 4/18 patients had viral encephalitis before diagnosis with anti‐NMDAR‐AE. One patient had a history of hepatocarcinoma surgery 8 years prior, and no relapse was reported during imaging. A total of in 1/7 women had ovarian teratomas when screened with PET/CT. A total of 1/7 of women had ovarian endometriosis diagnosed by pelvic MRI+.

### 3.2 Analysis on ^18^F‐FDG‐PET imaging and by visual assessment on MRI

Abnormal PET metabolic patterns were found in 18/18 (100%) patients, and 10/18 (55.56%) and 10/16 (62.5%) patients using MRI and EEG, respectively. Therefore, the sensitivity rate of PET/CT for discriminating brain lesions in patients with NMDAR‐AE was higher than that of MRI and EEG using visual assessment (Table 2). In 22.22% (4/18) patients, the regions of MRI abnormality were consistent with hypometabolic regions on FDG‐PET/CT (pat.3, pat.12, pat.17, and pat.18).

**TABLE 2:** Comparison of abnormal findings with visual assessment between PET, MRI, and EEG.

Pat.	Time of CSF^a^	CSF ab titer 1:	Serum ab titer 1:	Time of PET^b^	FDG PET/CT visual assessment		Time of MRI^c^	MRI hyperintensities and location	Time of EEG^d^	Abnormal awake EEG
					Hypermetabolism	Hypometabolism				
1	35	1 month: 3.2	1 month: ‐	45	Right cerebral hemisphere, left cerebellum, and left posterior cingulate	Left frontal and left temporo‐parietal lobe	44	Bilateral frontal lobes, semi‐oval centers, and lateral ventricles	36	Y
2	104	3 months: 1 5‐month: 1 8‐month: 1	3 months: 32 5‐month: 10 8‐month: 32	109	Bilateral cerebellum	Left anterior cingulate and left frontal lobe	112	‐	112	N
3	52	<1 month: ‐ 2 months: 100 4 months: 10 12 months: 1	<1 month: ‐ 2 months: ‐ 4 months: 10 12 months: ‐	109	Bilateral cerebellum	Left temporal lobe, left frontal lobe, and left anterior cingulate	57	Left temporal lobe, hippocampus, and basal ganglia	57	Y
4	91	3 months: 10 /AMPA1: 32 6 months: 1 /AMPA1: 100 10 months: 1 /AMPA1: 10	3 months: 100 /AMPA1:320 6 months: 10 /AMPA1: 320 10 months: ‐ /AMPA1: 32; 13 months: ‐ /AMPA1: 10 16 months:10 /AMPA1: 10	99	Bilateral basal ganglia and thalamus	Left temporo‐parietal lobe, left middle frontal lobe, and left post cingulate	96	‐	100	Y
5	39	1 month: 32 /GFAP: 3.2 7 months: ‐ /GFAP: ‐	1 month: 100 /GFAP: ‐ 7 months: 10 /GFAP: ‐	49	Suspicious of bilateral cerebellum	Bilateral occipital lobes, superior parietal lobe	40	Left anterior central gyrus cortex	41	Y
6	16	<1 month: 100 1 month: 3.2	<1 month: 100 1 month: 32	21	Left temporal lobe, bilateral basal ganglia, and right cerebellum	Left frontal and left parietal lobes	38	‐	9	Y
7	6	<1 month: 10 2 months: ‐	<1 month: ‐ 2 months: 10	8	Left superior temporal and right inferior temporal lobes	Left frontal and bilateral parietal lobes	8	Right parietal lobe	241	N
8	90	<1 month: ‐/CASPR2: /MOG: ‐ 3 months:1 /CASPR2:‐ /MOG:‐ 4 months:1 /CASPR2:‐ /MOG:‐	<1 month:‐/CASPR2:‐/MOG: 10 3 months:‐/CASPR2: 10 /MOG: 10 4 months:‐/CASPR2: 10 /MOG: ‐	100	Right cerebral hemisphere and left cerebellum	Left cerebral hemisphere, left thalamus, and right cerebellum	9	Left thalamus and pons		Not mentioned
9	2	<1 month: 3.2 /AMPAR2: 3.2	<1 month: 32 /AMPAR2: ‐ 4 months: 10 /AMPAR2: ‐; 7 months: 10 /AMPAR2: ‐	42	Right superior temporal lobe	Left frontal lobe and bilateral anterior cingulate cortex	5	‐	72	Y
10	59	2 months: 32	2 months: 100	82	‐	Bilateral superior parietal lobes	1	Left brachium pontis and left parietal lobe	64	Y
11	3	<1 month:100 /MOG: 3.2	<1 month: 10 /MOG: 10	11	Bilateral cerebellum and right temporal lobe	Bilateral occipital and left frontal–parietal lobes	4	‐	202	Y
12	47	<1 month: ‐ 2 months: 1 3 months: 1	<1 month: ‐ 2 months: ‐ 3 months: ‐	52	Right cerebellum	Right cerebral hemisphere and left cerebellum	8	Right temporal lobe	52	N
13	28	1 month: 32	‐	178	Bilateral basal ganglia	Bilateral frontal–parietal lobes	133	‐	130	N
14	12	<1 month: 10 2 months: ‐	<1 month: ‐ 2 months: ‐ 9 months: 10	30	Bilateral basal ganglia, thalamus, and left superior temporal lobe	Bilateral anterior cingulate cortex	6	Splenium of corpus callosum	55	Y
15	5	<1 month: 1	<1 month: 32	13	Bilateral basal ganglia and thalamus	Bilateral superior parietal and left middle frontal	5	‐	16	N
16	0	<1 month: 1000 7 months: 10	<1 month: 320 5 months: 1000 7 months: 320 10 months: 100	69	Bilateral basal ganglia and left superior temporal lobe	Bilateral anterior cingulate cortex	4	‐	0	N
17	50	2 months: 10	2 months: 1000	63	Left basal ganglia	Right frontal, parietal, temporal, left media frontal lobes, and left cerebellum	49	Bilateral temporal lobe, insular lobe, and lateral ventricle		Not mentioned
18	142	4 months: 1 /DNER:+ /anti‐Titin: + 7 months: 1 /anti‐Titin: +	4 months: 1000 /DNER:+ /anti‐Titin: + 5 months: 320 /DNER:+ /anti‐Titin: + 7 months: 1000 /anti‐Titin: +	162	‐	Right parietal‐occipital cortex	136	Abnormal signals in bilateral midline and parietal occipital cortex	139	Y

^a^ Time of CSF (days):The time from the onset of symptoms to cerebrospinal fluid puncture which the antibody of CSF is positive for the first time.

^b^ Time of PET (days):The time from the onset of symptoms to PET scan.

^c^ Time of MRI (days):The time from the onset of symptoms to MRI scan.

^d^ Time of EEG (days): The time from the onset of symptoms to EEG.

**FIGURE 1:**
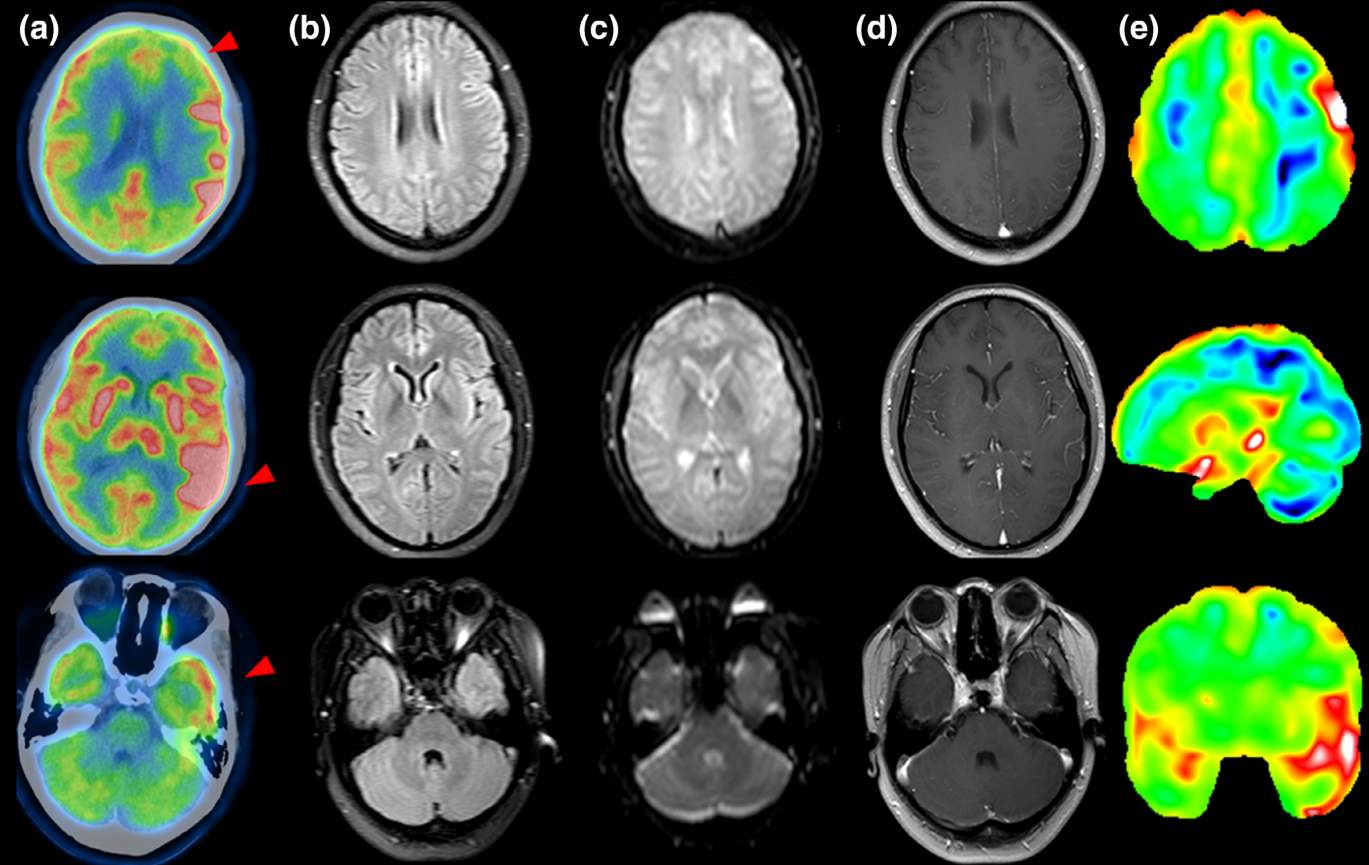
A typical case of the PET scan time is <1 month from symptom onset (pat. 6 had mental status changes and seizures for half a month, accompanied by cognitive impairment and speech disorders, and was diagnosed with ovarian teratoma). FDG‐PET in 21 days revealed that significant hypometabolism in left middle frontal, hypermetabolism in bilateral basal ganglia and left temporal lobe (A). However, cerebral MRI in 38 days shows unremarkable signal alterations on T2‐FLAIR image (B), DWI (C) and contrast enhanced MRI imaging (D). The FDG statistical deviation images analysis based on the *z*‐score (E).

**FIGURE 2:**
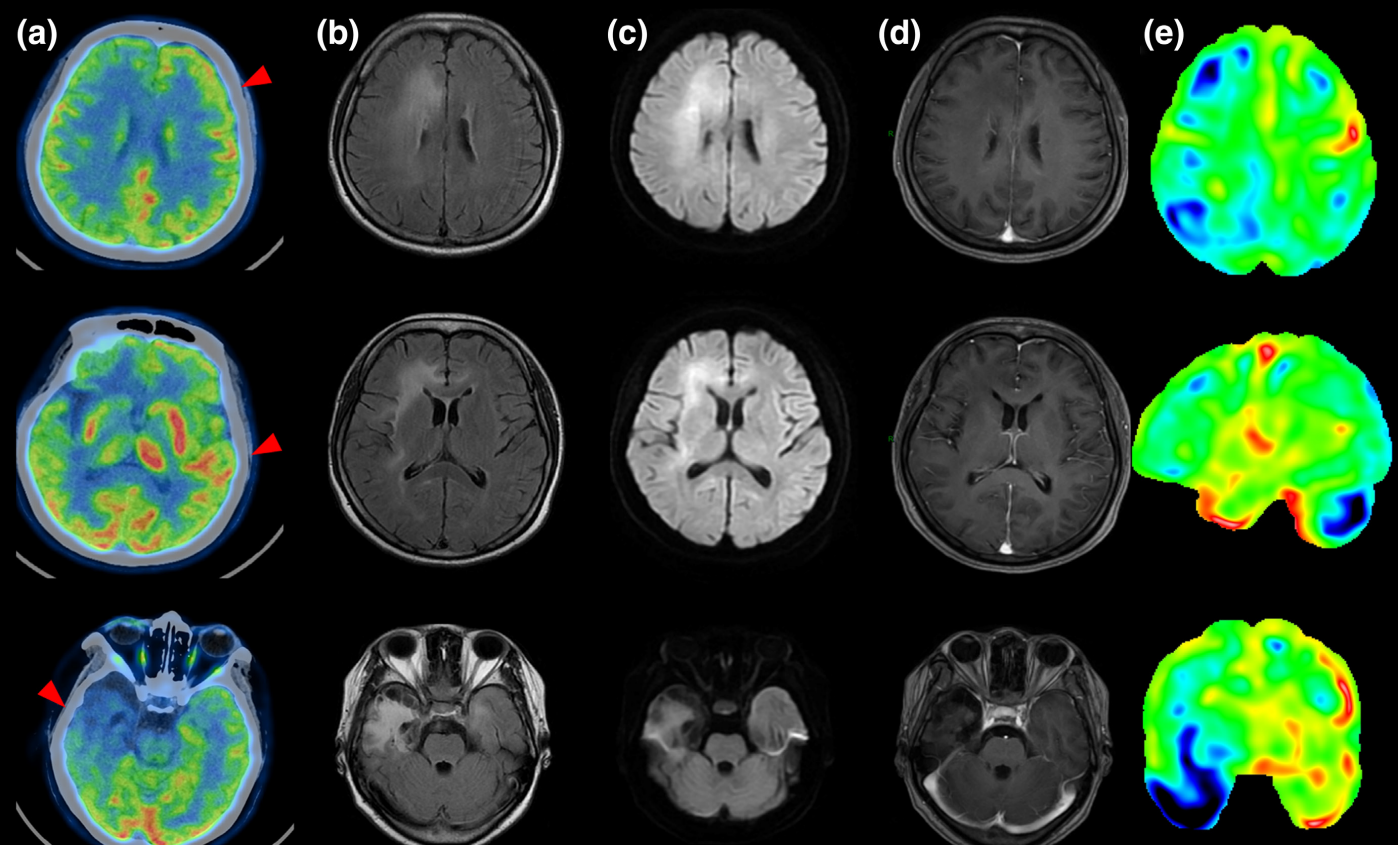
A typical case of the PET scan time is 2–3 months from symptom onset (pat. 17 had intermittent fever for 2 months, cognitive impairment, and speech disorders accompanied by abnormal behavior). FDG‐PET (A) at approximately 63 days revealed that hypermetabolism in left super temporal, hypometabolism in the left middle frontal and right frontal‐ parietal‐temporal lobes (especially in the right temporal lobe), which correspond to mild hyperintense in MRI areas. T2‐ FLAIR (B) and DWI (C) of axial MRI in 49 days showed multiple asymmetric white matter hyperintensities, especially in the right frontal‐temporal lobe, and lateral ventricle. There was a lack of enhancement in mentioned above areas of contrast‐enhanced MRI (D). The FDG statistical deviation images analysis based on the *z*‐score (E).

**FIGURE 3:**
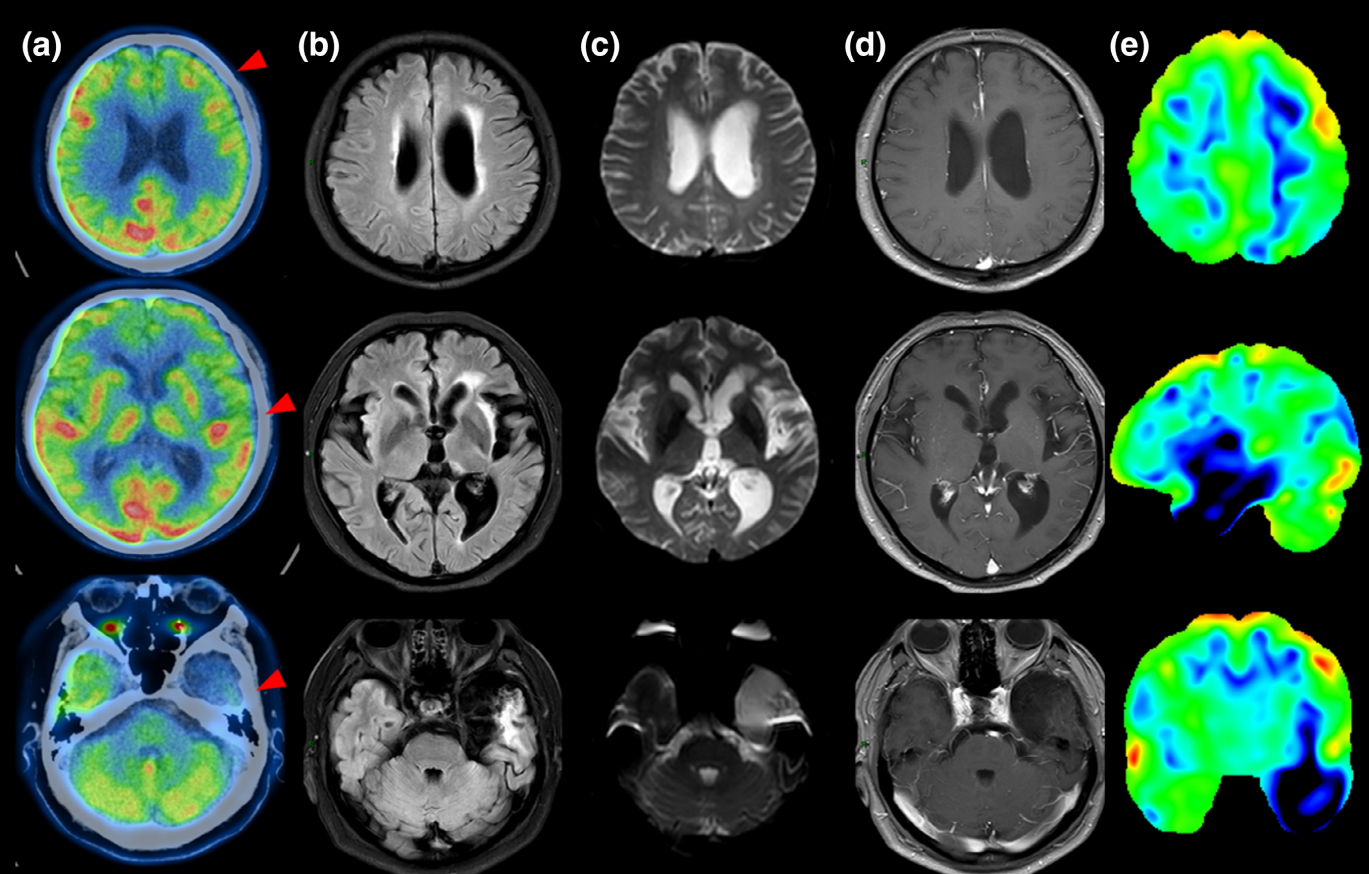
A typical case of the PET scan time for more than 3 months from the onset of symptoms (pat. 3 had intermittent fever with nausea, dizziness, headache, and cognitive impairment). FDG‐PET of 109 days revealed hypometabolism in the in the left middle frontal and left temporal lobe (A). T2‐FLAIR showed hyperintensities of axial MRI in the left temporal lobe, hippocampus, and insular lobe in 57 days (B). The left temporal lobe showed hyperintensities in DWI (C). There was no enhancement in mentioned above areas of contrast‐enhanced MRI (D). The FDG statistical deviation images analysis based on the *z*‐score (E).

Typical examples of examinations at different times are shown in Figures 1, 2, 3.

### 3.3 Regional analysis by software on ^18^F‐FDG‐PET imaging for all patients

We found significant hypometabolism in all patients in the frontal lobe, parietal lobe, cingulate gyrus, and occipital lobe (*t* = 2.75–5.24, *z* = 2.59–4.45, *p* < 0.001), and hypermetabolism in the cerebellum, basal ganglia, and para‐hippocampal gyrus (*T* = 2.88–5.04, *z* = 2.71–4.33) when data were compared to the age‐sex machine‐paired healthy‐control group using SPM with a height threshold of *p* < 0.005, *k* = 50 (Figure S2 shows the results of the SPM analysis).

When these results were compared with those using the NeuroQ software normal database, we found variable hyper and hypometabolic areas among patients. However, at least nine regions and at most 29 regions of abnormal metabolism in 47 different areas were identified in 18/18 (100%) patients. Because functional areas are vulnerable, results for the visual cortex and Broca's region should be considered with caution, and are not listed in our results. We found 18/18 patients in whom either of these regions was abnormal, including hypometabolic left middle/medial frontal cortex, hypometabolic anterior cingulate cortex, hypermetabolic superior temporal gyrus, hypermetabolic unilateral cerebellum or vermis, and hypermetabolic caudate nucleus. These results are shown in Table 3 and are similar to the SPM results. A typical example (pat.6) is shown in Figure S3.

**TABLE 3:** ROI analysis of FDG PET assessment by NeuroQ software compared to the normal database.

Stages	Hypermetabolism			Hypometabolism		
	Location	*n*	Percentage (%)	Location	*n*	Percentage (%)
All (*n* = 18)	*Unilateral super temporal cortex*	*11*	*61.11*	*Unilateral mid frontal cortex*	*14*	*77.78*
	Left sup temporal cortex	7	38.89	Left mid frontal cortex	12	66.67
	Right sup temporal cortex	8	44.44	Right mid frontal cortex	7	38.89
	*Unilateral basal ganglia*	*9*	*50.00*	*Unilateral medial frontal cortex*	*13*	*72.22*
	Unilateral caudate nucleus	8	44.44	Left medial frontal cortex	11	61.11
	Unilateral lentiform nucleus	5	22.22	Right medial frontal cortex	8	44.44
	*Cerebellum or vermis*	*10*	*55.56*	*Unilateral parietotemporal cortex*	*13*	*72.22*
	Vermis	8	44.44	Left parietotemporal cortex	11	61.11
	Left cerebellum	7	38.89	Right parietotemporal cortex	4	22.22
	Right cerebellum	5	27.78	*Unilateral ant cingulate cortex*	*9*	*50.00*
				Left ant cingulate cortex	8	44.44
				Right ant cingulate cortex	7	38.89
1‐month (*n* = 5)	*Unilateral super temporal cortex*	*4*	*80.00*	*Left mid/medial frontal cortex*	*4*	*80.00*
	Left sup temporal cortex	3	60.00	Left mid frontal cortex	4	80.00
	Right sup temporal cortex	3	60.00	Left medial frontal cortex	2	40.00
	*Unilateral basal ganglia*	*3*	*60.00*	*Left parietotemporal cortex*	*4*	*80.00*
	Unilateral caudate nucleus	3	60.00	*Unilateral ant cingulate cortex*	*1*	*20.00*
	Unilateral lentiform nucleus	1	20.00			
	*Left cerebellum*	*1*	*20.00*			
2‐3 months (*n* = 7)	*Unilateral super temporal cortex*	*5*	*71.43*	*Left mid/medial frontal cortex*	*5*	*71.43*
	Left sup temporal cortex	4	57.14	Left mid frontal cortex	3	42.86
	Right sup temporal cortex	3	42.86	Left medial frontal cortex	5	71.43
	*Unilateral basal ganglia*	*3*	*42.86*	*Unilateral ant cingulate cortex*	*4*	*57.14*
	Unilateral caudate nucleus	2	28.57	Left ant cingulate cortex	3	42.86
	Unilateral lentiform nucleus	2	28.57	Right ant cingulate cortex	3	42.86
	*Unilateral cerebellum or vermis*	*4*	*57.14*	*Left parietotemporal cortex*	*3*	*42.86*
	Vermis	3	42.86			
	Left cerebellum	3	42.86			
	Right cerebellum	2	28.57			
3 months (*n* = 6)	*Unilateral super lat temporal cortex*	*2*	*33.33*	*Left frontal mid/medial cortex*	*5*	*83.33*
	Left sup temporal cortex	0	0	Left mid frontal cortex	5	83.33
	Right super lat temporal cortex	2	33.33	Left medial frontal cortex	4	66.67
	*Unilateral basal ganglia*	*3*	*50.00*	*Unilateral ant Cingulate cortex*	*4*	*66.67*
	Unilateral caudate nucleus	3	50.00	Left ant cingulate cortex	4	66.67
	Unilateral lentiform nucleus	2	33.33	Right ant cingulate cortex	3	50.00
	*Unilateral cerebellum or vermis*	*5*	*83.33*	*Left parietotemporal cortex*	*4*	*66.67*
	Vermis	5	83.33			
	Left cerebellum	3	50.00			
	Right cerebellum	3	50.00			

*Note*: Because the visual cortex and Broca's region are commonly affected by function, we do not list those regions and they should be interpreted with caution. Unilateral suggested no matter the left/right or both cortex involved in a patient, count only once (in italics).

### 3.4 Subgroup analysis of changes in regional glucose metabolism

Figure S4 shows brain regions with abnormal glucose metabolism in patients divided into three groups based on time for subgroup analysis (see Section 2 methods) with a height threshold of *p* < 0.005, *k* = 50. These data were compared by SPM with paired healthy‐control groups. First, some regions of the frontal cortex and parietal lobe metabolism decreased during all three stages (*T* = 3.55–8.74, *z* = 2.67–4.23), and the cingulate metabolism also joined decreased between 2–3 months and persisted for >3 months (*T* = 4.14–8.48, *z* = 3.20–4.49). The metabolism of the lingual gyrus of the occipital lobe only decreased between 2–3 months (*T* = 3.21–3.78, *z* = 2.68–3.01). Second, we found significant hypermetabolism in the superior temporal gyrus, basal ganglia, thalamus, and cerebellum in the first month (*T* = 3.77–8.17, *z* = 2.78–4.12). Only the metabolism of the superior temporal gyrus increased between 2–3 months (*T* = 5.45, *z* = 3.80).

The brain regions with abnormal glucose metabolism compared with the NeuroQ normal database are shown in Table 3. The highest hypometabolism sensitivity rate was 83.33% (5/6) of patients in the left middle frontal cortex at >3 months. When extended to any region of the frontal lobe, both showed hypometabolism in 4/5, 7/7, and 5/6 patients over time. Additionally, the relatively high sensitivity rate for hypermetabolism involved the unilateral superior temporal cortex in 4/5 (80%) patients at <1 month and the cerebellum or vermis in 5/6 (83.33%) patients at >3 months. When extended to any region of the temporal lobe, 4/5 (80.0%), 7/7 (100%), and 3/6 (50.0%) patients experienced changes over time.

According to the sub‐analysis by the time of FDG PET, MRI and EEG abnormalities in any region occurred in 2/5 (40%), 5/7 (71.43%), and 3/6 (50%), and 3/5 (60%), 4/6 (66.67%) and 3/5 (60%) patients, respectively. The EEG report of pat.8 and pat.17 were not available.

### 3.5 Subgroup analysis of changes on MRI and EEG

Considering that patients did not undergo all same examination at the three stages, we listed the specific times for each examination in Table 2.

Data analyses were also grouped by MRI examination time although this grouping may have contributed to some study limitations. We found 5/9 (55.56%) patients with abnormal MRI findings in the first month, 4/5 (80%) between 2 and 3 months, and only 1/4 (25%) at >3 months. Single‐region MRI analysis showed that the unilateral temporal lobe (the most involved region) was abnormal in 3/10 (30.0%) patients at all stages, and the highest abnormal rate was found in only 2/5 (40.0%) patients between 2 and 3 months in the left temporal lobe.

Only 1/3 (33.33%) patients had an abnormal EEG in the first month, 6/7 (85.71%) between 2 and 3 months, and 3/6 (50%) at >3 months.

### 3.6 Analysis of changes for relapsed patients

Figure S5 shows brain regions with abnormal glucose metabolism in relapsed patients with a height threshold of *p* < 0.005, *k* = 50. These data were compared by SPM with paired healthy‐control groups. some regions of the temporal cortex metabolism increased (*T* = 5.15–12.54, *z* = 3.07–4.23), and the frontal metabolism decreased (*T* = 5.67–13.03, *z* = 3.22–4.37). When compared with the NeuroQ normal database, left caudate nucleus and unilateral superior temporal cortical hypermetabolism were more evident in 3/4 patients, and so was the left middle frontal cortical hypometabolism. Only 2/4 (50%) patients had an abnormal EEG and MRI.

## 4 DISCUSSION

Abnormal FDG‐PET brain scans have been reported in anti‐NMDAR encephalitis patients but metabolic patterns have been inconsistent across different regions.^4^, ^5^, ^6^, ^7^, ^8^, ^9^, ^10^, ^11^, ^12^, ^13^, ^14^, ^15^, ^16^, ^17^, ^18^, ^19^, ^20^, ^25^, ^27^, ^28^, ^29^, ^30^ In this study, we investigated the application of FDG‐PET during different stages of anti‐NMDAR encephalitis disease progression. First, notably (but unsurprisingly) we found that consistent with other diseases, the brain‐glucose metabolic patterns changed during different stages (*p* < 0.05).^10^, ^31^, ^32^ Second, hypometabolism of the middle/medial frontal cortex was common to all stages in our study (14/18 and 13/18 patients, respectively), although not all of them. The results were consistent with other studies on MRI,^33^, ^34^, ^35^, ^36^, ^37^ which reported decreased gray matter volume and reduced cerebral regional connectivity in left middle/medial frontal gyrus, they may have significantly correlated with memory performance and verbal inhibition control in NMDAR encephalitis. Moreover, according to our previous research on the cerebral metabolic network,^26^ the hub nodes are mainly located in the right frontal lobe in anti‐NMDAR encephalitis instead of in the left frontal lobe, as it is in healthy controls. We propose that hypometabolism of the left middle or medial frontal cortex may imply impaired neurons and that the right frontal lobe is a compensatory change. Third, we found that hypermetabolism of the superior temporal gyrus was more evident in the first 3 months (9/12 patients). Miao A. also found increased cerebral blood flow at the peak stage of the disease,^38^ but the gray matter volume was decreased in the left superior temporal gyrus on MRI.^37^ The functional connectivity between the frontal and temporal cortex by the dorsal caudate was reported in the literature,^39^ which may involve in the pathological mechanism of anti‐NMDAR encephalitis. However, this hypothesis requires future confirmation with larger scale data.

Our results may have potential implications for diagnosis and clinical management in the future. Notably, they may be useful in assisting in the clinical diagnosis of individualization, especially when time intervals vary between cases. For example, in our study, the temporal lobe in pat.6 was hypermetabolic and initially diagnosed in <1 month, but pat.3 already had a hypometabolic temporal lobe in the chronic stages when he was hospitalized. Additionally, PET can play a role in the early initiation of corticosteroid treatment that is effective for damage to the blood–brain barrier,^40^ especially when preliminary laboratory results and MRI are not initially positive. For instance, in this study the serum results of pat.7 were negative and MRI was ambiguous, but at the same stage, PET was obviously abnormal. These results were later confirmed by CSF analysis 5 days later. Furthermore, our study found that the later the therapeutic intervention, the more likely the patient was to relapse. Three of four patients who relapsed were treated after 3 months from symptom onset and relapsed 3–6 months later. Another patient was treated at 45 days and relapsed at 14 months. These data imply that earlier treatment results in a better prognosis. Moreover, our results were similar to those of other studies in general,^6^, ^7^, ^9^, ^11^, ^25^, ^27^, ^28^, ^29^, ^30^ but were inconsistent in detail. We propose that this is due to the differences between PET intervals in each report. Clearly, attention must be paid to the scan time, recognition of time‐varying changes and the common manifestations in FDG‐PET performance is important and helpful to clinicians in decision‐making.

Our study also revealed similar clinically relevant changes in MRI and EEG results in patients with anti‐NMDAR encephalitis. However, consistent with previous reports, FDG‐PET performance was superior.^4^, ^5^, ^6^, ^7^, ^8^, ^9^, ^18^ Furthermore, we found that MRI and EEG sensitivity rates were best in the 2–3‐month stage in our studies (80.0% for MRI and 85.71% for EEG). Therefore, early or chronically negative MRI and EEG results cannot rule out the presence of the disease. These results have important implications for the auxiliary diagnosis and clinical management of anti‐NMDAR encephalitis, especially in the early stages.

This study had several limitations. First, due to the retrospective design, the number of included cases was small, especially in the subgroup analyses. Moreover, three patient images were reconstructed using a different algorithm (TOF OSEM) from the other 15 patients (TrueX OSEM) and possibly should have been removed from the analysis. However, we included them in this article because the TOF reconstruction can produce higher voxel intensity and increased sensitivity versus the TrueX algorithm, and only a limited number in each subgroup was used for statistical analysis. Since studies on the clinical practice of using PET/CT scans for NMDAR were limited, and the time was often missing, we could not validate the data by review. Therefore, prospective clinical trials are warranted. Second, Probasco et al.^7^ showed that occipital hypometabolism was clinically significant in anti‐NMDAR encephalitis, similar to the SPM results in our study. However, the occipital lobes and Broca's region are commonly affected by function and external conditions; therefore, we do not list those regions by patient in our study because we could have underestimated the results. Third, because of the therapeutic regimen and duration of individual differences, we did not evaluate treatment effects on PET images. However, we listed the time and will include therapy in future studies. This study aimed to evaluate the effects over time in patients with anti‐NMADR antibodies by using PET/CT, MRI, and EEG, but to overcome the abovementioned limitations, increased sample sizes are required in future studies.

In conclusion, this study suggested that hypometabolism of the middle or media frontal cortex could be a common metabolic pattern over the disease course, whereas the metabolism of other regions and the performance of MRI and EEG reflected time‐varying changes associated with progression. The sensitivity rate of FDG‐PET was superior to that of MRI and EEG. These results have potential value for the diagnosis and clinical management of patients with anti‐NMDAR‐AE.

## CONFLICT OF INTEREST

The authors declare that they have no conflict of interest.

## PATIENT CONSENT STATEMENT

The requirement for obtaining informed consent was waived.

## Supporting information


Figures S1‐S5

## Data Availability

Data generated or analyzed during the study are available from the corresponding author by request.
