# Supplementary material for: Hypometabolism of the left middle/medial frontal lobe on FDG‐PET in anti–NMDA receptor encephalitis: Comparison with MRI and EEG findings
Source: CNS Neurosci Ther. 2023 Feb 23;29(6):1624–35. doi: 10.1111/cns.14125 (PMC10173717; doi:10.1111/cns.14125)
Supplement: Supplementary file 1 — Figures S1‐S5 [file CNS-29-1624-s001.docx]

Supplemental Figures Captions

Supplemental Figure 1. Flow diagram shows the enrollment and exclusion criteria of the patients


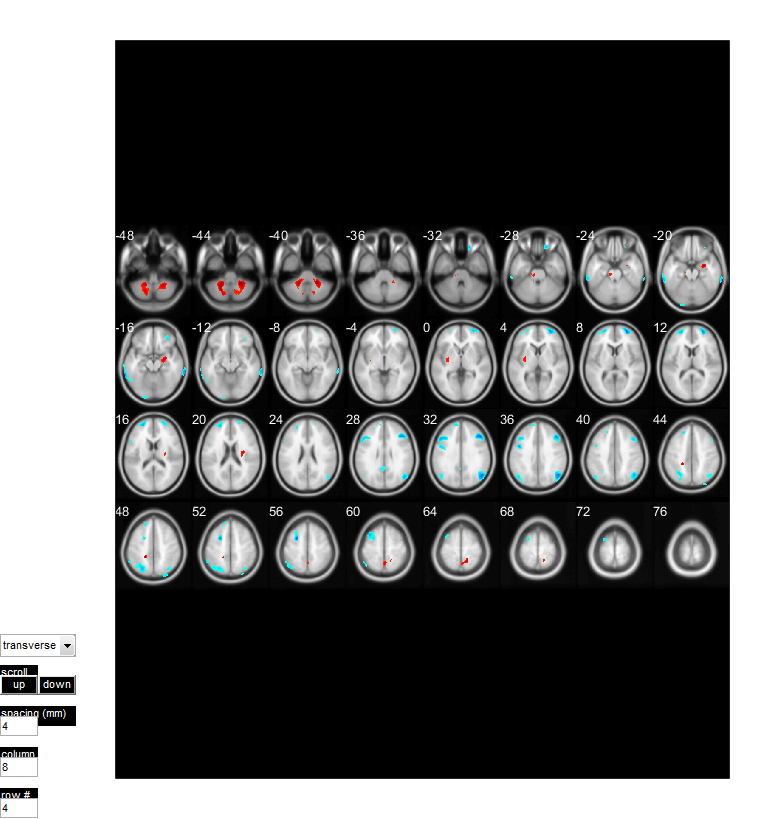


Supplemental Figure 2. SPM analysis of FDG uptake pattern on superimposed MRI, with red marks corresponding to hypermetabolism and blue marks to hypometabolism compared with the age-gender-machine paired healthy control group.


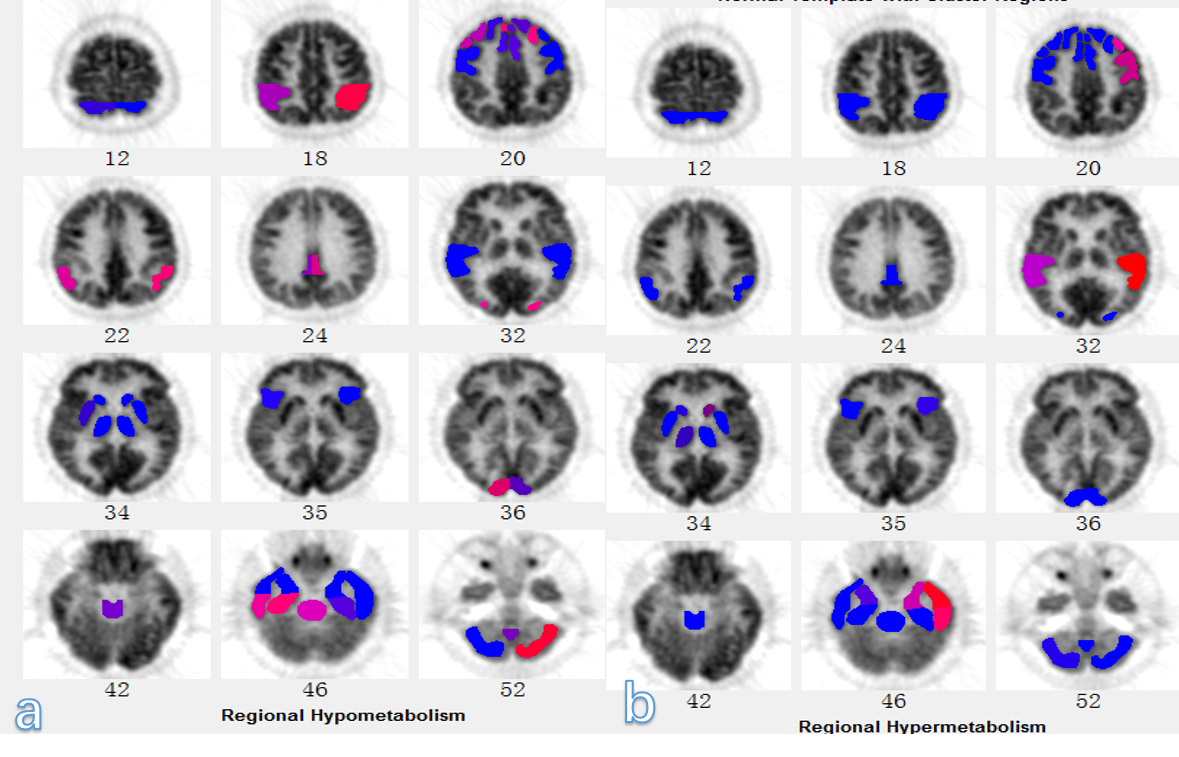


Supplemental Figure 3. Example of individual analyses (pat. 6) by NeuroQ (a for hypermetabolism and b for hypometabolism), with red marks corresponding to the highest z score and blue to the lowest z score.


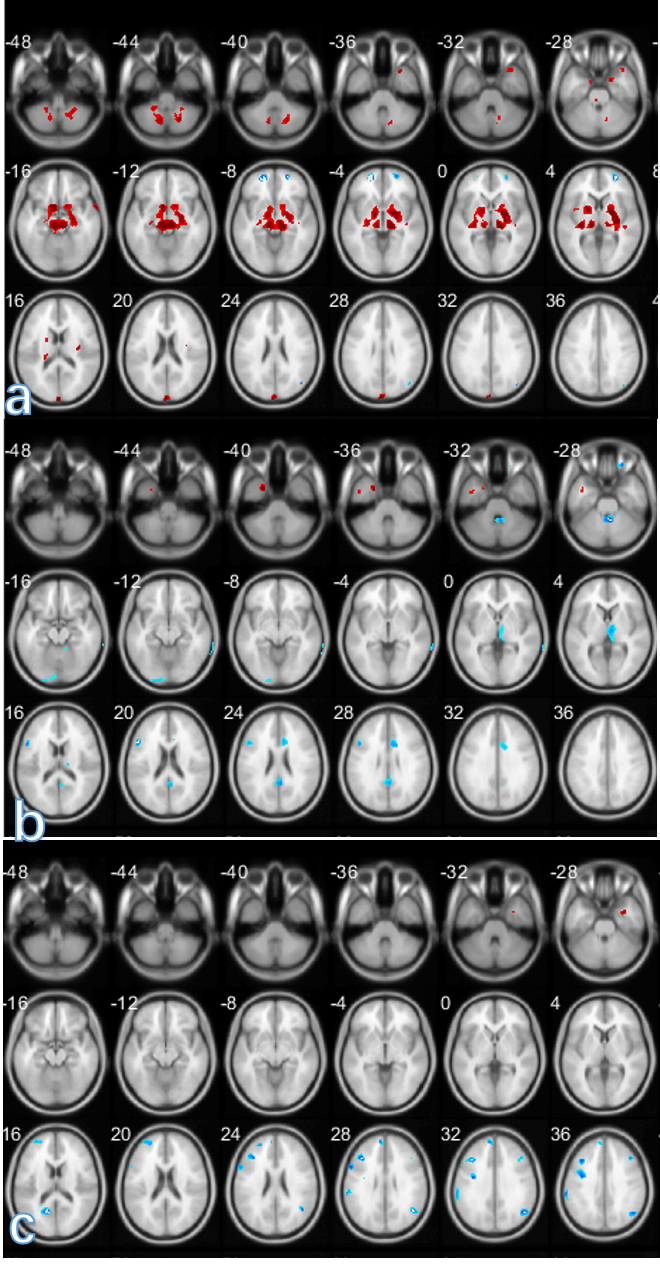


Supplemental Figure 4. SPM analysis of FDG uptake pattern showed on superimposed MRI for different stages, with red marks corresponding to hypermetabolism and blue marks to hypometabolism compared with the age-gender-machine paired healthy control group (a for acute stage, b for subacute stage, and c for chronic stage).


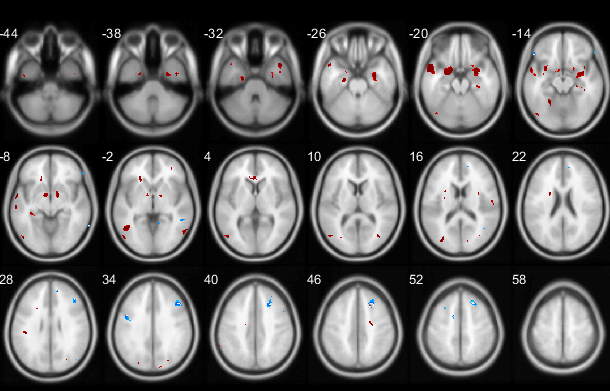
 Supplemental Figure 5. SPM analysis of FDG uptake pattern for relapsed patients showed on superimposed MRI, with red marks corresponding to hypermetabolism and blue marks to hypometabolism compared with the age-gender-machine paired healthy control group.
